# Enhancing Learner Participation in Online Discussion Forums in Massive Open Online Courses: The Role of Mandatory Participation

**DOI:** 10.3389/fpsyg.2022.819640

**Published:** 2022-04-15

**Authors:** Zhao Du, Fang Wang, Shan Wang, Xiao Xiao

**Affiliations:** ^1^Business School of Sport, Beijing Sport University, Beijing, China; ^2^Lazaridis School of Business and Economics, Wilfrid Laurier University, Waterloo, ON, Canada; ^3^Department of Finance and Management Science, University of Saskatchewan, Saskatoon, SK, Canada; ^4^Higher Education Press, Beijing, China

**Keywords:** MOOC, online education, online discussion forum, mandatory participation, voluntary participation, learner characteristics, learning performance

## Abstract

Online discussion forums are an essential and standard setup in online courses to facilitate interactions among learners. However, learners’ inadequate participation in online discussion forums is a long-standing challenge, which necessitates instructor intervention and the design consideration of online learning platforms. This research proposes and studies the role of mandatory participation, i.e., learners’ participation in online course forums by instructors’ requirements, in fostering their voluntary participation and boosting their learning performance. This novel effect link between mandatory participation and voluntary participation has not been assessed in previous research. An empirical study is conducted using a large-scale dataset of 27,767 learners from a leading massive open online course (MOOC) platform in China. The findings indicate that besides its direct effect on learning performance, learners’ mandatory participation has a significant positive effect on their voluntary participation in online course forums, enhancing learning performance. Moreover, the effect of mandatory participation on voluntary participation varies across learner groups, being more prominent for early registrants than late registrants and part-time learners than full-time learners. This research contributes to the online learning literature by introducing mandatory participation as a viable approach to foster voluntary participation and boost learning performance through enhanced voluntary participation. It provides evidence on the effectiveness of the novel design feature of MOOC platforms that enables and facilitates the mandatory participation mechanism in online learning.

## Introduction

Online asynchronous discussion forums are a popular tool to facilitate learner interactions. It is among the most widely used tools to support traditional in-class teaching and student-centered learning (Garrison et al., 2000; Davies and Graff, 2005; Garrison, 2007; Watson et al., 2016; Covelli, 2017). It is a standard setup in online learning and on massive open online course (MOOC) platforms (Chiu and Hew, 2018). Participating in online forum discussions is beneficial to learners and can significantly enhance their learning experience and boost their performance (Kamboj and Rahman, 2017; Almatrafi and Johri, 2018; Chiu and Hew, 2018).

However, the lack of learner participation in online course forums is a prevalent and concerning issue facing instructors and online course platforms. In most cases, a fragment of the learner population participates in online course forums, and their participation is often shallow and insufficient (Wise et al., 2013; Sharif and Magrill, 2015). Prior research has studied learners’ participation in online course forums by examining various individual, social, pedagogical, and technological factors that affect learner participation (Deng and Tavares, 2013) and investigating different ways to enhance it. These include instructor involvement (Zhang et al., 2018), student facilitators (Hew and Cheung, 2008), scripting (i.e., designing rules that specify and sequence interactions, roles, and activities), and summarizing (Peterson and Roseth, 2016). Yet, as low learner participation continues to challenge online learning, online education stakeholders look for new approaches for enhancing learner participation in online course forums.

This study explores a promising way of enhancing learners’ voluntary participation by embedding mandatory participation activities in online courses. Mandatory participation refers to learners’ participation in online course forums in response to instructor requirements. Conversely, voluntary participation is learners’ participation in online course forums at their will (Brown et al., 2002). Online instructors can easily design and implement instructional components for learners’ mandatory participation. For example, instructors may post questions in course videos and require learners to answer and discuss them in online course forums.

To support such an instructional component, a leading MOOC platform in China has implemented a novel design feature of online course forums that separates a mandatory participation section and a voluntary participation section for learner interactions. The mandatory participation section is for learners to post their answers and discussions on questions that are embedded in course videos. Learners’ participation in this section is monitored and graded by instructors. The voluntary participation section is for learners to interact and discuss topics of their interest and choice. In such a setting of both mandatory and voluntary participation, if mandatory participation can foster voluntary participation, instructors can strategically use the mandatory participation mechanism to enhance learners’ voluntary participation and boost learning performance. However, to the best of our knowledge, no research has examined the effect link between mandatory participation and voluntary participation.

The proposition on the effect of mandatory participation on voluntary participation is rooted in information systems (IS) adoption/acceptance literature and the self-determination theory (SDT). Prior IS research suggests that mandated information technology (IT) use can stimulate compliant and engaged user responses (Bhattacherjee et al., 2018). That is, mandated use may induce voluntary use behavior. However, IS research attributes mandated use and volitional use to two separate IT usage environments, and does not explicitly study the potential link between them. In a similar vein, SDT differentiates controlled and self-determined (i.e., voluntary) behaviors, and suggests that the process of internalization can transform behaviors motivated by external contingencies to those internally motivated (Deci and Ryan, 1985; Deci et al., 1991). As recent IT designs increasingly incorporate both mandatory and voluntary use functions, in which settings mandatory activities can be implemented for user behavior intervention, understanding the potential link between mandatory and voluntary participations is imperative and meaningful.

In view of the above, this research proposes and examines the effect of mandatory participation in online course forums on learners’ voluntary participation and learning performance. This research is driven by the need for enhancing learners’ voluntary participation in online course forums as well as scant research on the effect link between mandatory and voluntary participations. Mandatory participation and voluntary participation are learner activities of different motivations and nature. However, because mandatory participation engages learners in and familiarizes them with online course forums, it may enhance their voluntary participation and boost learner performance through enhanced voluntary participation. Particularly, this research inquiries into the following research questions:

RQ1: Does mandatory participation affect learners’ voluntary participation in online course forums?
RQ2: Does and how does the effect of mandatory participation on voluntary participation vary across learner characteristics?

An empirical study analyses a dataset from 27,767 learners of a MOOC. Results suggest that mandatory participation significantly enhances learners’ voluntary participation in online course forums, leading to better learning outcomes. The positive effect of mandatory participation on voluntary participation varies across learner groups of registration timing and work status, being more prominent for early registrants than late registrants and learners with a full-time job than those without.

This research contributes to the literature in two ways. First, it adds to the online education literature by proposing and studying mandatory participation as a useful and viable instructional design for enhancing voluntary participation and suggesting the heterogeneity of the effect across various learner groups. The literature of online education continues to stress the value and challenge of enhancing learners’ voluntary participation in online course forums and recommend new approaches for this purpose (Peterson and Roseth, 2016). Proposing and studying the role of mandatory participation as an instructional design that is capable of enhancing voluntary participation provides a new avenue to understand and promote learners’ voluntary participation in online course forums. Second, this study extends the IS research on mandated IT use to the context of online education, where mandatory and voluntary uses of IT functions co-exist. Traditional IS research studies mandated use and volitional use of IT as mutually exclusive (Karahanna et al., 1999; Bhattacherjee et al., 2018). The relationship between mandatory participation and voluntary participation examined in this research not only advances the research on IT adoption and acceptance, but better fits the usage design of modern IS that is modularized and provides high affordances.

The findings of this study derive actionable recommendations to online instructors and online course platforms. Designing course elements for mandatory participation is a useful approach for online instructors to enhance learners’ voluntary participation in online course forums. Thus, instructors can incorporate it as a viable teaching strategy. In addition, the effectiveness of mandatory participation on fostering voluntary participation varies across various learner characteristics. Therefore, to decide the optimal course strategy, instructors need to pay attention to the characteristics of learners. Online course platforms can provide design features and assessment measures to support instructors’ evaluation of the effectiveness of mandatory participation.

## Literature Review

### Online Learning and Online Forum Participation

Online forums are among the most popular tools to facilitate communications and interactions among instructors and learners (Garrison et al., 2000; Garrison, 2007). It has been widely used to support traditional offline instruction and acts as a standard component for online course setup. In particular, due to the lack of face-to-face interaction in the traditional classroom setting, online forums are considered a great supplementary tool to enhance the interactions among instructors and students and promote collaborative learning in online courses (Ludvigsen and Mørch, 2010; Singh and Mørch, 2018).

Two streams of literature on online forums in the education context are relevant to this study. The first stream of literature studies various factors that affect learners’ online forum participation. Specifically, they examined the roles of instructors (Mazzolini and Maddison, 2003, 2007; Andresen, 2009; Zhang et al., 2018), student facilitators (Hew and Cheung, 2008), individual factors, social factors, pedagogical factors, technological factors (Deng and Tavares, 2013), and computer-supported cooperative learning strategies (Peterson and Roseth, 2016) in fostering learner participation. For example, Du et al. (2022) suggest that instructors can strategically use their posting and replying activities on online course forums to interact with learners to enhance their learning experience. Peterson and Roseth (2016) suggest that learning strategies such as social interdependence, summarizing, scripts, and synchronicity can enhance learner participation in online forums.

The second stream of literature investigates whether online forum participation benefits learning performance. Most studies report a positive effect of online forum participation on learning performance. For example, the number of learner activities in online course forums, such as posting and post viewing, is positively associated with their learning performance (Morris et al., 2005; Bonafini et al., 2017) and negatively associated with the dropout rate (Balakrishnan and Coetzee, 2013). On the other hand, some scholars suggest that the absence of non-verbal social cues in online forum participation diminishes communication quality and impedes social relations, thus may harm learning performance (Peterson and Roseth, 2016). Overall, online course forum participation is beneficial in learners’ experience. Online course platforms and instructors could benefit from new approaches that can help enhance learner engagement and learning outcomes.

### Mandated and Volitional Information Technology Use

Mandated use and volitional us are two types of IT uses investigated in the IS literature (Karahanna et al., 1999; Zolkepli and Kamarulzaman, 2015; Bhattacherjee et al., 2018). The IS research has considered mandated use and volitional use as two separate IT adoption/use environments and studied the differences in user experiences between them (Karahanna et al., 1999; Bhattacherjee et al., 2018). In the volitional use environments, users perceive the technology adoption or the decision to use as their willful choices. However, in the mandated use environments, users are mostly reactive because the use of IT artifacts is institutionally compulsory (Brown et al., 2002; Chae and Poole, 2005).

Extant studies have indicated that user attitudes and acceptance toward IT in mandated use environments differ from those in volitional IT use environments (Brown et al., 2002). Instead of perceived usefulness as the major driver of IT use in the volitional environment, ease of use serves as the primary determinant of users’ behavioral intention in mandated use (Brown et al., 2002). Furthermore, the effects of social influence (Venkatesh et al., 2003) and subjective norms (Hartwick and Barki, 1994) are more prominent in mandated use than volitional use. In particular, the effect of social influence on users’ behavioral intention is stronger in the early stage of a mandated use (Venkatesh et al., 2003).

Although the investigation of IT use is rich in the IS literature, none has addressed the relationship between mandated and volitional IT uses because they are featured in mutually exclusive IT use environments. The social media literature and the education literature on MOOCs have predominantly studied voluntary participation (Zolkepli and Kamarulzaman, 2015; Wang and Wang, 2020; Farivar et al., 2021; Du et al., 2022). As the mixed mandatory and voluntary use of IT functionalities is increasingly popular in IT designs, studying the effect of mandatory use on voluntary use is imperative and meaningful.

### Self-Determination Theory and Its Application in Education Research

SDT is an influential theory about the motivation behind people’s choice and behavior, and is a common lens to explore student motivation and volitional involvement in learning (Deci and Ryan, 1985; Deci et al., 1991). SDT distinguishes between self-determined and controlled types of intention and behavior (Deci et al., 1991; Chen and Jang, 2010). Self-determined behaviors are volitional, enforced by one’s sense of self, and regulated by choice. Controlled behaviors are compelled by interpersonal or intrapsychic force and regulated by compliance (Deci and Ryan, 1985; Deci et al., 1991). These concepts correspond to the voluntary and mandatory participations studied in this research.

SDT specifies three basic psychological needs that motivate self-initiated behaviors: autonomy, competence, and relatedness (Deci and Ryan, 1985; Deci et al., 1991). Autonomy refers to “being self-initiating and self-regulating of one’s actions” (p. 327, Deci et al., 1991). Competence involves “understanding how to attain various external and internal outcomes and being efficacious in performing the requisite actions” (p. 327, Deci et al., 1991). Relatedness involves “developing secure and satisfying connections with others in one’s social milieu” (p. 327, Deci et al., 1991). SDT views internalization as a motivated and proactive process through which regulation by external contingencies can become internal and no longer require external contingencies (Deci and Ryan, 1985; Deci et al., 1991). That is, controlled or mandatory behaviors can transform into volitional or voluntary behaviors through internalization.

SDT has been widely applied in education research to study student motivation and examine approaches to enhance student involvement and engagement in learning (Chen and Jang, 2010; Butz and Stupnisky, 2017; Vasconcellos et al., 2020). For example, Filak and Nicolini (2018) report that students perceive lower levels of autonomy, competence, and relatedness in online courses than in face-to-face courses. This may explain the low-level student engagement in online courses (Sharif and Magrill, 2015; Almatrafi and Johri, 2018). Butz and Stupnisky (2017) suggest implementing an online discussion board intervention to scaffold the development of students’ feelings of relatedness (i.e., feeling connected to others) to improve student success in online courses.

While SDT differentiates controlled and self-determined behaviors and has been widely applied to study the effectiveness of instructional strategies in boosting students’ learning engagement, extant research has rarely examined the effects of controlled behavior on voluntary behavior, but rather treats them as two ends of a continuum (Chen and Jang, 2010). Different from previous studies, this research connects the two by considering mandatory participation in online course forums as a potentially effective instructional strategy to boost learners’ voluntary participation.

## Research Model

**FIGURE 1:**
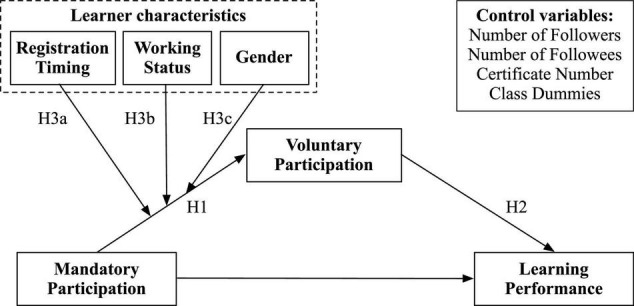
Research model.

Building on the online education literature and drawing from SDT, this research examines the effect of mandatory participation on learners’ voluntary participation in online course forums and subsequently their academic performance. As presented in Figure 1, we posit that mandatory participation, in addition to its direct effect on learner performance, can foster voluntary participation in online course forums and indirectly boost learner performance. The effect of mandatory participation on voluntary participation may vary across learner characteristics such as gender, registration timing, and working status.

### Effect of Mandatory Participation

SDT suggests that fulfillment of autonomy, competence, and relatedness can increase volitional behavior (Deci and Ryan, 1985; Deci et al., 1991). Accordingly, we argue that mandatory participation can foster voluntary participation in multiple ways. First, mandatory participation creates awareness of and draws learners to online course forums. Through mandatory participation, learners familiarize themselves with engagement activities in online course forums (Stein et al., 2015). As learners overcome the initial hurdle of participation and become used to participation, they are more likely to continue their online discussions through voluntary participation.

Second, mandatory participation can increase learners’ competence in online forum participation (Deci and Ryan, 1985), resulting in enhanced voluntary participation. Mandatory participation provides learners the opportunities to experience and appraise the benefits from and control over participating in online course forums (Brown et al., 2002). When learners experience benefits and positive social interactions from online forum participation, they will be more likely to continue their involvement through voluntary participation.

Third, mandatory participation can increase learners’ relatedness to peers (Deci and Ryan, 1985), fostering voluntary participation in online course forums. Learners interact with instructors and peers through mandatory participation in online course forums, which fosters a sense of community among the instructors and learners. The sense of community is critical for promoting their future voluntary participation (Luo et al., 2017; Cacciamani et al., 2019). For example, Luo et al. (2017) have shown that interactions can strengthen learners’ sense of community, which affects their stickiness on and the continuous use intention of e-learning platforms. Based on the above, we posit:

H1: *Learners’ mandatory participation in online course forums positively affects their voluntary participation in online course forums.*

Voluntary participation in online course forums can help learners improve their course performance. Compared with watching video lectures, voluntary participation in online course forums offers the benefits of social learning from peers. First, from the perspective of knowledge acquisition, voluntary participation in online course forums enables a collaborative learning process (Singh and Mørch, 2018). Learners can ask questions based on their interests and difficulties and obtain answers from peers. In peer discussions, learners may share their knowledge and interpretations of course contents based on their work and life experiences. Their sharing, in turn, can be beneficial for improving the understanding of course contents (Sun et al., 2018). The collaborative knowledge construction process helps learners develop a better understanding of course contents. Second, from a psychological perspective, participating in online course forums keeps learners engaged with course contents and connected with peers. Engagement with course contents and connection with peers are both critical in a disengaged online learning environment. Thus, participating in online discussions helps to keep learners motivated in their learning process and enhances the learning results (Balakrishnan and Coetzee, 2013). Based on the above, we posit:

H2: *Learners’ voluntary participation in online course forums positively affects their learning performance.*

### Moderation Effects

The effect of mandatory participation on fostering voluntary participation in online course forums may not be consistent across learners. Rather, depending on learners’ varied motivations for and perceived benefits from online forum participation, the effect of mandatory participation on voluntary participation may differ. Thus, we hypothesize and study the heterogeneous effect of mandatory participation on voluntary participation across three learner characteristics: registration timing, working status, and gender.

As discussed in the education literature, course registration timing is a behavioral indicator of learners (Ford et al., 2008; Tompkins et al., 2019). Early registrants who register for a course before class starts often have a good working habit of non-procrastination and conscientiousness (Ford et al., 2008). Conscientious learners are hardworking, energetic, and persevering, more likely to achieve academic success in both traditional and online course settings (Costa and McCrae, 1992; Tompkins et al., 2019). Later registrants who register after a course starts, on the other hand, are often observed with less performance than those who register early (Smith et al., 2002; Shriner, 2014). As early registrants may be more hardworking and ambitious, they may be more likely to identify and grasp all possible learning opportunities. When they benefit from mandatory participation, they may be more likely to continue engaging with online discussion through voluntary participation to maximize their learning opportunity and outcome. Based on the above, we posit:

H3a: *The effect of mandatory participation on voluntary participation varies across learners with different registration timing. The effect is stronger for early registrants of a course than for late registrants.*

The education literature also notes the difference between learners of different working status. Learners with an ongoing full-time job have time constraints, so they constantly look for efficient ways of learning (McKenzie and Gow, 2004; MacCann et al., 2012; Tompkins et al., 2019). Participation in online course forums offers the opportunity to learn from peers. It may help learners quickly identify common issues in learning and also continuously motivate them to study. Learners with a full-time job may highly value these benefits due to their time and other learning constraints. Consequently, when these learners engage in and benefit from mandatory participation in online forums, they are more likely to continue with online discussion through voluntary participation. Based on the above arguments, we posit:

H3b: *The effect of mandatory participation on voluntary participation varies across learners of different working statuses. The effect is stronger for learners with full-time jobs than for those without.*

In addition, the education literature has considered gender-based differences in learning, such as gender-based differences in motivation, perception, communication behaviors, and study habits in learning (Price, 2006; Yukselturk and Bulut, 2009). Male and female learners often prefer different learning strategies (Park et al., 2019). For example, although women are more socially interactive (Murphy et al., 1997; Hunter et al., 2005; Ma, 2015), they often participate less in online forum discussions and need a special effort to be engaged (Hakkarainen and Palonen, 2003; Chan and Chan, 2011; Davis, 2021). Comparatively, males are more active online and are not particularly in need of intervention to be engaged (Chan and Chan, 2011). Based on the above, we posit:

H3c: *The effect of mandatory participation on voluntary participation varies across learners’ genders. The effect is stronger for female learners than for male learners.*

## Data and Variables

### Data

We obtained a large-scale proprietary dataset from a leading MOOC platform in China. The platform was launched in 2004. As of the end of 2019, it has provided 21,465 sessions of 8,302 courses to the public. The majority of the MOOCs on the platform are produced and instructed by faculties from prestigious colleges and universities in China. Courses are organized into 12 categories, including agriculture, medicine, history, philosophy, engineering, pedagogy, law, science, management, economics, literature, and art. The majority of the courses are of the undergraduate level.

Similar to those on United States platforms such as Coursera and edX, MOOCs on this platform are video-based and are set up with online course forums for learner interactions. The unique feature of this platform is that the online course forums consist of three sub-forums, Course Discussion, Q&A, and General Discussion, each designated to a specific purpose. The Course Discussion sub-forum is for learners to answer and discuss instructors’ questions embedded in course videos or posted in the forum. This is a mandatory participation sub-forum, monitored by instructors for evaluating student participation. Voluntary participation sub-forums include the sub-forum of Q&A and the one of General Discussion. The Q&A sub-forum is for learners to post, at their will, questions and comments that are relevant to the course contents. Either the instructors or other learners may address these questions. The sub-forum on General Discussion is for learners to discuss any topics that may or may not be related to course content.

Our dataset consists of fine-grained learner-level data from the MOOC course “The essence of C programming language.” The course had been offered seven times (i.e., has seven course sessions) between November 2014 and June 2018. The durations of the course sessions range from 17 to 21 weeks, and the claimed course loads for these sessions range from 1 to 3 h per week. A total of 218,292 learners registered in the seven sessions of the course. Of these learners, 190,525 were dropped from analysis because there is no course performance data. These include 20,413 learners who registered to a session after its closure, and 170,112 learners who registered before the closure of a session but did not engage in any graded learning activities. The final dataset for analysis consists of 27,767 records of learners with non-zero scores (14% of the total registrants). The high dropout rate and low completion rate are typical in MOOCs and are consistent with prior findings in literature (Rivard, 2013; Chen et al., 2020).

### Variables

Table 1 presents the definitions and operationalization of the main variables in this study. Because this research utilizes secondary data, data availability is considered in variable and measurement selections.

**TABLE 1:** Variable definitions and operationalization.

Variable	Definition and operationalization
Performance	The academic performance of a learner in a course session. It is measured by the final score of a learner in a course session.
MandatoryPart	The mandatory participation of a learner in a course session. It is measured by the number of posts that a learner has contributed in the online course forum in response to instructor requirements.
VoluntaryPart	The voluntary participation of a learner in a course session. It is measured by the number of posts that a learner has voluntarily contributed in the online course forum (i.e., number of total posts – number of mandatory posts).
RegisTime	The registration timing of a learner in a course session. It is operationalized as a dummy variable indicating whether a learner registered for a course session before its start (1 = Yes, 0 = No).
WorkStatus	The working status of a learner. It is operationalized as a dummy variable indicating whether a learner had a full-time job while taking a course (1 = Yes, 0 = No).
Gender	The gender of a learner. It is operationalized as a dummy variable (1 = Female, 0 = Non-female).
Follows	The number of learners that a learner followed on the MOOC platform when he/she registered for a course.
Fans	The number of fans that a learner had on the MOOC platform when he/she registered for a course.
CertNum	The number of course certificates that a learner had received when he/she registered for a course.

The dependant variable in this study is academic performance of a learner in a class (*Performance*). It is operationalized as the final score a learner has earned in a course. The main variables under study are mandatory participation (*MandatoryPart*) and voluntary participation (*VoluntaryPart*). *MandatoryPart* is operationalized as the number of participating activities of a learner in response to instructor requirements. Particularly in our study context, it is measured by the number of posts a learner contributed in the mandatory participation section of the online forum in response to instructor questions raised in course videos. *VoluntaryPart* is operationalized by subtracting the number of mandatory participating activities from the total number of activities of a learner in the online course forum.

To investigate the heterogeneous effect of mandatory participation on voluntary participation across learners with different characteristics, we include three dummy variables as moderators. The dummy variable *RegisTime* measures whether a learner registered for a course before its start. *WorkStatus* refers to whether a learner had a full-time job while taking a course. The dummy variable *Gender* denotes the gender of a learner.

Based on the literature on e-learning, we include two social networking variables and one learning experience variable as the control variables. Social networking variables include the number of users that a focal learner follows on the MOOC platform (*Follows*), and the number of fans a learner has on the MOOC platform (*Fans*). Learning experience is controlled by the number of course certificates that a learner received before registering for the course (*CertNum*). Furthermore, we also include course session dummies to control for the unobserved heterogeneity across course sessions.

### Descriptive Statistics

Tables 2, 3 present the descriptive statistics and correlations of the variables. The average final course score of learners in our sample is 30.57 out of a total score of 100. A perusal of the score data reveals that among the 27,767 learners in our sample, only 7,064 (25%) earned a score higher than the passing grade (i.e., 60/100). The low average score suggests a low commitment from MOOC learners to participate in required learning activities. The average numbers of mandatory participation and voluntary participation in online course forums are 6.19 and 1.30, respectively. That is, mandatory participation in online course forums accounts for a large portion (82.65%) of online course forum participation of learners. The mean of registration timing is 0.084. That is, the majority of learners in our sample (91.6%) registered for a course after it started. This is different from offline courses in the traditional settings in which most students register for a course before classes start. The mean of working status is 0.034, which means that most learners in our sample (96.6%) are college students or learners who do not have full-time jobs. The mean of gender is 0.097. That is, a large portion of the learners in our sample (90.3%) are male. This is consistent with the fact that more male learners are interested in taking programming courses. The average numbers of followed users and fans of learners in our sample in the MOOC platform are 0.074 and 0.063, respectively. The low averages of social networking measures reveal that learners have limited social connections in the MOOC platform. The average number of prior course certificates is 0.286. That is, most learners are novices who are not experienced in MOOC learning.

**TABLE 2:** Descriptive statistics.

Variable	Obs.	Mean	Std. Dev.	Min.	Median	Max.
Performance	27,767	30.571	31.283	0.060	14	100
MandatoryPart	27,767	6.194	20.146	0	0	208
VoluntaryPart	27,767	1.301	11.517	0	0	686
RegisTime	27,767	0.084	0.278	0	0	1
WorkStatus	27,767	0.034	0.182	0	0	1
Gender	27,767	0.097	0.296	0	0	1
Follows	27,767	0.074	1.210	0	0	102
Fans	27,767	0.063	3.164	0	0	306
CertNum	27,767	0.286	7.007	0	0	361

**TABLE 3:** Correlations.

No.	Variables	1	2	3	4	5	6	7	8	9
1	Performance	1.000								
2	MandatoryPart	0.406*	1.000							
3	VoluntaryPart	0.117*	0.147*	1.000						
4	RegisTime	−0.087*	−0.032*	0.003	1.000					
5	WorkStatus	−0.023*	–0.003	0.012*	0.051*	1.000				
6	Gender	0.099*	0.042*	0.029*	–0.002	0.009*	1.000			
7	Follows	–0.001	–0.000	0.003	0.007*	0.008*	0.012*	1.000		
8	Fans	0.007*	0.001	0.003	0.001	0.015*	0.012*	0.126*	1.000	
9	CertNum	0.025*	0.005*	0.018*	0.004*	0.033*	0.023*	0.052*	0.325*	1.000

*p < 0.1.

### Empirical Model

To test the moderated mediation effects using an integrated approach, we follow the suggested method by Preacher and Hayes (2008) and Hayes (2017). The hypotheses are tested by Equations (1, 2) using the bootstrapping procedure with 5,000 iterations. A natural logarithm transformation is taken for the variables that are highly skewed, such as mandatory participation and voluntary participation. In Equations (1, 2), a learner’s academic performance in a course (*Performance*_*i*_) is the dependent variable, the natural logarithm of the mandatory participation in the online course forum (*Log#MandatoryPart_*i*_*) is the independent variable, and the natural logarithm of the voluntary participation in the online course forum (*Log#VoluntaryPart_*i*_*) is the mediator.


(1) $$\begin{array}{c} \text{Log}\,\#\,{\text{VoluntaryPart}}_{1}=\\ \beta_{0}+\beta_{1}\,\text{Log}\,\#\,{\text{MandatoryPart}}_{i}+\beta_{2}\,{\text{RegisTime}}_{i}\\ +\beta_{3}\,{\text{WorkStatus}}_{i}+\beta_{4}\,{\text{Gender}}_{i}\\ +\beta_{5}\,\text{Log}\,\#\,{\text{MandatoryPart}}_{i}\times {\text{RegisTime}}_{i}\\ +\beta_{6}\,\text{Log}\,\#\,{\text{MandatoryPart}}_{i}\times {\text{WorkStatus}}_{i}\\ +\beta_{7}\,\text{Log}\,\#\,{\text{MandatoryPart}}_{i}\times {\text{Gender}}_{i}+\delta \,\varphi_{i}+\mu_{i} \end{array}$$

(2) $$\begin{array}{c} {\mathrm{Performance}}_{i}={\text{a}}_{0}+{\text{a}}_{1}\,\text{Log}\,\#\,{\text{MandatoryPart}}_{i}+\\ a_{2}\,\text{Log}\,\#\,{\text{VoluntaryPart}}_{i}+{\text{a}}_{3}\,{\text{RegisTime}}_{i}+\\ a_{4}\,{\text{WorkStatus}}_{i}+{\text{a}}_{5}\,{\text{Gender}}_{i}+\gamma \,\varphi_{i}+\varepsilon_{i} \end{array}$$

Specifically, the empirical analyses consist of two steps. First, to test whether voluntary participation mediates the impact of mandatory participation on learner performance, we conduct a mediation analysis by excluding the three interaction terms of *MandatoryPart*_*i*_ with the moderators in Equation (1). Then, to test the moderation effect of registration timing, working status, and gender on the effect of mandatory participation on voluntary participation of online course forums, we include the three interaction terms of *MandatoryPart*_*i*_ with the moderators in Equation (1). Furthermore, φ_*i*_ denotes the set of control variables in this study, including *Follows*_*i*_, *Fans*_*i*_, *CertNum*_*i*_, and course session dummies.

## Results

**FIGURE 2:**
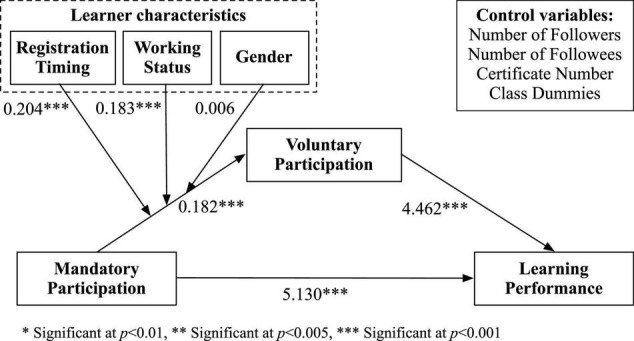
Estimation results of the moderated mediation model.

The estimation results are summarized in Figure 2 and explained in the following sections.

### Testing the Mediation Effect

Table 4 presents the results of the mediation analysis. The parameter estimations of the main variables in Equations (1, 2) are reported in Models A and B, respectively, in Table 4. Shown in Model A of Table 4, learners’ mandatory participation in online course forums has a significant positive impact on their voluntary participation (β= 0.209, *p <* 0.001). Meanwhile, shown in Model B of Table 4, learners’ mandatory participation and voluntary participation in online course forums have significant positive impacts on their course performance (β_*MandatoryPart*_ = 5.171, β_*VoluntoryPart*_ = 4.156, *p <* 0.001). These results indicate that learners’ mandatory participation in online course forums has a significant positive impact on their learning performance. This effect is partially mediated by their voluntary participation in online course forums. That is, in addition to its direct effect on learning performance, mandatory participation fosters voluntary participation, which adds to learning performance.

**TABLE 4:** Mediation model (*n* = 27,767).

Predictor	Coef.	S.E.	*t*-value	*p*-value
**Model A: Mediator variable model (Voluntary participation)**				
Log#MandatoryPart	0.209	0.003	66.930	0.000
**Model B: Dependent variable model (Learner performance)**				
Log#MandatoryPart	5.171	0.157	32.870	0.000
Log#VoluntaryPart	4.156	0.280	14.810	0.000

Table 5 presents the results of the bias-corrected direct and indirect effects of mandatory participation on learner performance. The 95% bootstrap confidence interval of the indirect effect of mandatory participation on course performance (i.e., via voluntary participation) is [0.729, 1.008], which does not include zero. This result confirms those reported in Table 4, indicating that mandatory participation in online course forums has a significantly positive effect on learners’ course performance through the mediation of voluntary participation. In particular, the mediation effect accounts for 14.39% [i.e., 0.869/(0.869 + 5.171)] of the total effect. H1 and H2 are supported.

**TABLE 5:** Bias corrected indirect effect of the mandatory participation on learner performance (*n* = 27,767).

Predictor	Estimate	Bootstrap S.E.	Bootstrap confidence interval
Log#MandatoryPart (direct)	5.171	0.151	[4.875, 5.466]
Log#MandatoryPart (indirect)	0.869	0.071	[0.729, 1.008]

*The number of bootstrap samples for bias-corrected bootstrap confidence intervals is 5,000 times. The confidence level for all confidence intervals in the output is 95%.*

### Testing the Moderated Mediation Effect

Table 6 presents the results of the moderated mediation analysis. The results confirm the findings reported in section “Testing the Mediation Effect,” i.e., the partial mediation effect of learners’ voluntary participation in online course forums on the relationship between mandatory participation and learner performance. Moreover, reported in Model A of Table 6, *RegisTime* and *WorkStatus* significantly moderate the effects of learners’ mandatory participation on their voluntary participation in online course forums. The estimation of the interaction term *Log#MandatoryPart × RegisTime* (β= 0.204) is significantly positive, indicating that the effect of mandatory participation on voluntary participation is more prominent for early registrants than late registrants. Thus, H3a is supported. The estimation of the interaction term *Log#MandatoryPart × WorkStatus* (β= 0.183) is significantly positive, indicating that the effect of mandatory participation on voluntary participation is more prominent for part-time learners (i.e., learners with full-time jobs) than full-time learners. H3b is supported. However, the estimation result of the interaction term *Log#MandatoryPart × Gender* is not significant, indicating that the effect of mandatory participation on voluntary participation does not differ by gender. Thus, H3c is not supported.

**TABLE 6:** Moderated mediation model (*n* = 27,767) (single moderation).

Predictor	Coef.	S.E.	*t*-value	*p*-value
**Model A: Mediator variable model (Voluntary participation)**				
Log#MandatoryPart	0.182	0.003	53.180	0.000
RegisTime	–0.003	0.016	–0.190	0.851
WorkStatus	0.017	0.024	0.730	0.467
Gender	0.031	0.014	2.300	0.022
Log#MandatoryPart × RegisTime	0.204	0.012	17.710	0.000
Log#MandatoryPart × WorkStatus	0.183	0.016	11.220	0.000
Log#MandatoryPart × Gender	0.006	0.009	0.660	0.506
**Model B: Dependent variable model (Learner performance)**				
Log#MandatoryPart	5.130	0.156	30.930	0.000
Log#VoluntaryPart	4.462	0.279	16.010	0.000
RegisTime	–8.334	0.637	–13.090	0.000
WorkStatus	–7.824	0.935	–8.360	0.000
Gender	9.840	0.567	17.340	0.000

Table 7 presents the bias-corrected direct and indirect effects of mandatory participation on learner performance and the moderation effects of *RegisTime*, *WorkStatus*, and *Gender*. For all parameter estimations, zero does not fall in the 95% bootstrap confidence intervals. The results confirm the mediation effect of voluntary participation between mandatory participation and learner performance reported in Table 6. The mediation effect accounts for a significantly larger portion of the total effect when learners are early registrants (23.69% for early registrants vs. 14.45% for late registrants) and part-time learners (23.51% for part-time learners vs. 14.45% for full-time learners).

**TABLE 7:** Bias corrected indirect effect of mandatory participation on learner performance and the moderation effects (*n* = 27,767).

Predictor	Moderator	Estimate	Bootstrap S.E.	Bootstrap confidence interval
Log#MandatoryPart (direct)		5.080	0.153	[4.781, 5.379]
Log#MandatoryPart (indirect)	RegisTime (= 1)	1.821	0.178	[1.473, 2.169]
	RegisTime (= 0)	0.858	0.067	[0.727, 0.989]
	WorkStatus (= 1)	1.721	0.210	[1.310, 2.131]
	WorkStatus (= 0)	0.858	0.067	[0.727, 0.989]
	Gender (= 1)	0.884	0.089	[0.709, 1.060]
	Gender (= 0)	0.858	0.067	[0.727, 0.989]

*The number of bootstrap samples for bias-corrected bootstrap confidence intervals is 5,000 times. The confidence level for all confidence intervals in output is 95%.*

## Discussion and Conclusion

This research studies the role of mandatory participation in fostering learners’ voluntary participation in online course forums, which affects their learning performance in online courses. An empirical study on a dataset of 27,767 learners from a leading MOOC platform in China indicates that mandatory participation has a direct and an indirect effect on learners’ course performance. In its indirect effect, mandatory participation fosters learners’ voluntary participation in online course forums, which contributes to better learner performance. In addition, the effect of mandatory participation on voluntary participation varies across learner characteristics, being more prominent for early registrants than late registrants and part-time learners than full-time learners. No difference between gender groups is found.

### Theoretical Contributions

This research contributes to the online learning literature (Henrie et al., 2015; Du et al., 2021) by introducing mandatory participation of online course forums as a viable approach to foster voluntary participation in online course forums and boost learning performance. Low learner participation continues to challenge online education. Despite the commonly recognized benefits of participating in online course forums, learners do not participate much (Sharif and Magrill, 2015; Almatrafi and Johri, 2018). The lack of voluntary participation is evident in our data. Specifically, the number of mandatory participation activities is more than four times that of voluntary participation activities. To motivate learner participation, online education research has studied many approaches, such as instructor involvement (Zhang et al., 2018) and student facilitators (Hew and Cheung, 2008), and continues looking for new viable ways (Peterson and Roseth, 2016). This study proposes mandatory participation as a feasible instructional design for enhancing voluntary participation. The effect of mandatory participation on voluntary participation is explained through SDT (Deci and Ryan, 1985; Deci et al., 1991) and empirically validated. This research adds to the education literature and provides a new avenue to understand and promote learners’ online course forum participation.

In addition, this research explores and provides evidence on the heterogeneity of the effect of mandatory participation across different learner groups. Education research has long stressed individual differences in learning tactics and performance (Price, 2006; Park et al., 2019). The results on the differential effects of mandatory participation on voluntary participation across learners with different registration timing and working status are in line with discussions in previous research (MacCann et al., 2012; Tompkins et al., 2019). These results stress the importance of a targeted instructional strategy.

This study also contributes to the IS literature by studying mandatory and voluntary participations in a co-existent setting and in the context of online education. It draws from the traditional IT adoption/acceptance research in which mandated and volitional IT uses are two different technology use environments (Venkatesh et al., 2003; Hwang et al., 2016; Bhattacherjee et al., 2018) and develops the research to a new scenario where mandatory and voluntary uses of IT functions co-exist. As the usage design of modern IS is modularized and provides high affordances, the coexistence of mandatory and voluntary use functions is now prevalent. To the best of our knowledge, no prior research has investigated the relationship between mandatory use and voluntary use of IT functionalities. Therefore, this study provides a new perspective for IS researchers to gain insight into user participation and the synergies between mandatory participation and voluntary participation in online course forums.

### Practical Contributions

This research offers actionable practical guidelines to online course instructors and platforms. For instructors, this research suggests that mandatory participation is a viable approach for encouraging voluntary participation of learners in online course forums. Instructors can incorporate course components that include mandatory participation in the course syllabus to foster learners’ voluntary participation. In addition, the effectiveness of mandatory participation on fostering voluntary participation varies across certain learner characteristics. Therefore, instructors need to pay attention to the characteristics of learners to decide the optimal course strategy. For example, for classes with more early-registered students or part-time learners, instructors can post more questions for mandatory participation or increase the percentage of mandatory participation evaluation in the overall assessment. By this way, instructors can effectively motivate and achieve enhanced voluntary learning efforts.

The findings of this research also suggest that online course platforms incorporate design features for supporting instructors’ course component of mandatory participation. Indeed, this research is partially motivated by a novel design feature implemented in a leading MOOC platform in China, which separates the mandatory participation and voluntary participation sections in online course forums to facilitate learners’ responses to embedded questions in course videos. While embedding questions in course videos is designed mainly to engage learners in video contents, the subsequent requirements on learners’ responses not only lead to their mandatory participation in online course forums but also enhance their voluntary participation. This research provides evidence on the effectiveness of the novel design feature for mandatory learner participation in online learning. In addition, as mandatory participation and voluntary participation co-exist but may need different supports on activities and assessments, platform designers could fine-tune their offerings to provide customized supports to users.

### Limitations and Further Research

Several limitations of this research inform further research. First, the empirical study of this research is carried out with a sample of 27,767 learners from seven sessions of a course between November 2014 and June 2018. Selecting the sample from one course is due to the large data volume on MOOCs. While the sample is sufficient for our study, future research could employ samples from courses of other categories or samples of multiple courses to account for the differences across course categories and ensure the generalizability of the results.

Second, the empirical study of this research is based on secondary data. As such, variable selection in this study is constrained by data availability. For this reason, learner characteristics such as registration timing, working status, and gender, instead of their personality and psychological factors, are included in the study to control for learner heterogeneity. Further research can seek and include learners’ personality and psychological factors to validate our results and identify further insights.

Third, three learner characteristics are examined as moderators of the mandatory-voluntary participation relationship. The effects of two moderators, i.e., registration timing and working status, are significant, and that of gender is not. The insignificance of gender as a moderator can be re-tested and confirmed. Our sample consists of learners of a programming course, in which the majority of learners are male. Learners from other types of courses can be studied, and survey and experimental research can be carried out to confirm our results and identify plausible explanations. In addition, further research could study additional moderators, especially learner personality and/or psychological factors.

## Data Availability Statement

The original contributions presented in the study are included in the article/supplementary material. Further inquiries can be directed to the corresponding authors.

## Ethics Statement

Ethical review and approval were not required for the study in accordance with the local legislation and institutional requirements. Written informed consent for participation was not required for this study in accordance with the national legislation and the institutional requirements.

## Author Contributions

ZD, FW, and SW designed the research. XX collected the data. ZD and SW conducted the data analysis. FW and ZD wrote the manuscript. All authors contributed to the article and approved the submitted version.

## Conflict of Interest

The authors declare that the research was conducted in the absence of any commercial or financial relationships that could be construed as a potential conflict of interest.

## Publisher’s Note

All claims expressed in this article are solely those of the authors and do not necessarily represent those of their affiliated organizations, or those of the publisher, the editors and the reviewers. Any product that may be evaluated in this article, or claim that may be made by its manufacturer, is not guaranteed or endorsed by the publisher.
